# Supplementary material for: Protocol to produce a systematic Arenavirus and Hantavirus host-pathogen database: Project ArHa
Source: Wellcome Open Res. 2025 Aug 6;10:227. Originally published 2025 Apr 28. [Version 2] doi: 10.12688/wellcomeopenres.24037.2 (PMC12415512; doi:10.12688/wellcomeopenres.24037.2)
Supplement: Peer review history

# Reviewer report: Reviewer response for version 2

**DOI:** 10.21956/wellcomeopenres.27303.r129419

**Published:** 2025-09-09

**Authors:** Richard Ostfeld

**Affiliations:** 1 Cary Institute of Ecosystem Studies, Millbrook, New York, USA

The paper describes a protocol to produce a database called “Project ArHa” that consists of published records of direct or indirect detections of Arenaviruses and Hantaviruses in rodents and shrews worldwide. Such a database organizes and curates published associations between these small mammal hosts and two epidemiologically important groups of viruses, each with zoonotic members.  The authors expect the database to be applied to studies of human disease risk, including the causes of zoonotic spillover and mitigation of disease outbreaks. Given the importance of rodents and shrews as hosts of numerous pathogens, including zoonotic viruses, such a database would help correct the current biased emphasis on other mammalian orders.

 The protocol includes detailed descriptions of the strategy for conducting literature searches, screening studies to facilitate decisions on inclusion, extracting the data from included studies, and processing the data before posting/publishing.  The study evinces considerable care in attending to many pitfalls inherent to the construction of similar databases addressing the occurrences of pathogens in hosts.  These pitfalls, well described in the paper, include issues such as how to interpret non-detections of a pathogen in a host, how to detect and address sampling bias and publication bias, and how to reconcile conflicting diagnostic methods and conclusions. Responding to the first round of review, the authors altered their search strategy and data processing protocols to further safeguard against incorporation of biases and address uncertainties in data quality.  I would expect the communities of disease ecologists and epidemiologists to pounce on the ArHa database, as has happened with a much more taxonomically diverse forebear, the Global Mammal Parasite Database (Stephens et al. 2017), which as of 14 August 2025 has been cited 122 times (Google Scholar statistic).   

 My major concern with the study is with the potential for inappropriate uses of the database.  On the one hand, once the well-curated database is available for use, misuses can hardly be blamed on the authors.  On the other hand, the authors could help prevent unfortunate applications by better addressing both the motivations for creating the database and limitations in its use. The paper repeatedly stresses improving understanding of risk of pathogen transmission and spillover. Indeed, the database seems designed specifically to stimulate studies to address potential “spillover hotspots”, their ecological causes, and possible interventions.  Unfortunately, the database consists of detections of hantaviruses or arenaviruses in particular small-mammal hosts; it does not address transmission of the viruses by those hosts. It is easy to misuse data on pathogen detection when prevalence (or seroprevalence) in a population or species of host does not reflect the magnitude of onward transmission (including spillover) by that population or species.  As suggested by recent literature in disease ecology, users of the database could easily conflate the two. The elephant shrew in the room is that mere detection of a pathogen in a host is not tantamount to that host being important in transmission of that pathogen to other hosts.  For example, Sin Nombre Virus has been detected in wild populations of *Mus musculus* and *Tamias minimus* in New Mexico at prevalences similar to that of *Peromyscus maniculatus*, but with no corresponding evidence that *M. musculus* or *T. minimus* can act as “reservoir hosts”, amplifying the virus or contributing to spillover transmission (Goodfellow et al. 2021). *Sciurus carolinensis *is frequently infected with the bacterial agent of Lyme disease, but its net effect is to reduce overall transmission risk of this tick-borne pathogen to humans (Keesing et al. 2009, Levi et al. 2016). Distinguishing between the mere presence of a virus (or antibodies to a virus) in a host population and the ability of that host to transmit the virus onward is perhaps beyond the scope of the database.  But I would suggest that the authors not miss the opportunity to include this as a key limitation of their effort. 

 I am concerned about the ability of the general search terms in Table 1 to retrieve the relevant literature. For instance, papers on squirrels, chipmunks, beavers, marmots, gophers, gerbils, jerboas, etc., might not be picked up with the search terms “rodent”, “rat”, and “mouse”.  I was confused about whether the database was intended to include only shrews within the eulipotyphlans or all eulipotyphlans. Although the term “insectivore” refers both to the Order Insectivora and a dietary category, use of this term might catch many relevant papers that would otherwise be missed.  “Small mammal” might be another important, general search term. I was also confused as to why mention of shrews as one of the key host taxa was dropped from the “Data extraction” section onwards.  I did not understand the insistence that the dataset would be “static”, suggesting that there would be no efforts to update it.  The care applied to the literature search, screening, extracting, and processing would seem to lend itself to a workflow that could be reapplied and updated at reasonable intervals, say every five years.

 The paper consists of an Introduction, Methods, and Discussion, but no Results. It seems that the authors intend for the community of users to generate worthy results based on their database and wish to devote detailed attention to their protocol to foster transparency and utility.  Nevertheless, a paper proposing the development of the database seems less useful than one that includes the database itself, or that uses the database to address patterns or hypotheses.

 References:

 [Reference 1], [Reference 2], [Reference 3], [Reference 4]

Is the study design appropriate for the research question?

Yes

Is the rationale for, and objectives of, the study clearly described?

Yes

Are sufficient details of the methods provided to allow replication by others?

Yes

Are the datasets clearly presented in a useable and accessible format?

Not applicable

Reviewer Expertise:

Community ecology, disease ecology, population ecology, biology of mammals

I confirm that I have read this submission and believe that I have an appropriate level of expertise to confirm that it is of an acceptable scientific standard, however I have significant reservations, as outlined above.

## References

[1] : Tracing Transmission of Sin Nombre Virus and Discovery of Infection in Multiple Rodent Species. *Journal of Virology* .2021;95(23) : 10.1128/JVI.01534-21 10.1128/JVI.01534-21 PMC857738734549977

[2] : Hosts as ecological traps for the vector of Lyme disease. *Proceedings of the Royal Society B: Biological Sciences* .2009;276(1675) : 10.1098/rspb.2009.1159 3911-3919 10.1098/rspb.2009.1159 PMC282578019692412

[3] : Quantifying dilution and amplification in a community of hosts for tick‐borne pathogens. *Ecological Applications* .2016;26(2) : 10.1890/15-0122 484-498 10.1890/15-0122 27209790

[4] : Global Mammal Parasite Database version 2.0. *Ecology* .2017;98(5) : 10.1002/ecy.1799 10.1002/ecy.1799 28273333

# Reviewer report: Reviewer response for version 2

**DOI:** 10.21956/wellcomeopenres.27303.r129413

**Published:** 2025-09-08

**Authors:** Phelipe Magalhães Duarte

**Affiliations:** 1 Federal Rural University of Pernambuco (UFRPE), Recife, Brazil

The manuscript, titled "Protocol to produce a systematic Arenavirus and Hantavirus host-pathogen database: Project ArHa," aimed to build a global, standardized, and relational dataset of arenavirus and hantavirus detections in wild-caught rodents and shrews, integrating ecological, epidemiological, and genomic information.

 The study uses appropriate language, is coherent, and is technically sound.

 The manuscript sequence is coherent and logical.

 The methodology is adequate.

 The results are based on scientific findings and are replicable.

 Suggestion:

 Keywords could be improved.

 The purpose of keywords is to increase the manuscript's reach in databases and through search methods based on the topic.

 I suggest including keywords in the references that are not in the title or abstract to increase reach.

Is the study design appropriate for the research question?

Yes

Is the rationale for, and objectives of, the study clearly described?

Yes

Are sufficient details of the methods provided to allow replication by others?

Yes

Are the datasets clearly presented in a useable and accessible format?

Yes

Reviewer Expertise:

virology, one health

I confirm that I have read this submission and believe that I have an appropriate level of expertise to confirm that it is of an acceptable scientific standard.

## References

[1] : The Hidden Threat: Rodent-Borne Viruses and Their Impact on Public Health. *Viruses* .2025;17(6) : 10.3390/v17060809 10.3390/v17060809 PMC1219736140573400

# Reviewer report: Reviewer response for version 1

**DOI:** 10.21956/wellcomeopenres.26520.r123245

**Published:** 2025-06-03

**Authors:** Roger Hewson

**Affiliations:** 1 London School of Hygiene & Tropical Medicine, London, UK

Summary: The manuscript presents a protocol for the development of ArHa, a unified, spatially and temporally explicit database synthesising published data on Arenaviruses and Hantaviruses in wild-caught rodents and shrews. These zoonotic viruses pose significant public health threats, yet their ecology and spillover risks remain under characterised, particularly in Africa and parts of Asia.

 The proposed ArHa dataset will collate and harmonise data on host sampling, pathogen detection, and genomic sequences from global literature using a systematic review approach. Key features include metadata on sampling effort, species identification, detection assays and geospatial resolution. This enables users to assess sampling bias, prevalence, and host-pathogen associations across space and time. The dataset will be developed in R, made openly accessible via GitHub and Zenodo and adhere to FAIR principles. It is designed to support predictive modelling, ecological research, and public health planning.

 The rationale for and objectives of, the study are well described and the manuscript articulates a strong rationale for the study, grounded in:

 - The zoonotic threat posed by arenaviruses and hantaviruses;

 - Sparse and fragmented ecological data, particularly in LMICs;

 - Increasing anthropogenic drivers of spillover (land-use change, biodiversity loss, climate change);

 - The need for integrated ecological and genomic data to support risk assessments and public health interventions.

 Objectives are clearly stated and include constructing a global, standardised, relational dataset; setting out  sampling effort and detection biases; linking rodent, pathogen and genomic data across time and geography and making sure data interoperability through taxonomic harmonisation and public release via FAIR-compliant platforms.

 The study design is appropriate and well aligned with objectives:

 - A systematic review approach using two major literature databases (PubMed and Web of Science);

 - Transparent inclusion/exclusion criteria, screening via Rayyan and documentation using a PRISMA flowchart;

 - Extraction of three linked datasets: small mammal sampling, pathogen testing results and genomic sequences;

 - Metadata capture on spatial resolution, sampling effort and methodological details (assay type, species identification);

 - Harmonisation of species names (via GBIF and NCBI) and pathogen names (via ICTV), ensures taxonomic consistency.

 The design is appropriate for developing existing literature into a coherent, workable 'data product' that can serve ecological, virological as well as public health purposes.

 The planned datasets are clearly presented and authors acknowledge limitations of the study such as data incompleteness, static nature of the dataset, and potential publication bias. To address these they propose mitigations like flagging imputed data and encouraging data deposition to GBIF).

 Scientific points to address

 While the objectives are described within the text,they could be more clearly listed in bullet or numbered format near the end of the introduction or start of the methods for clarity.

 It would be useful if authors could identify how ambiguous or inconsistent taxonomic identifications will be handled. Similarly the treatment of conflicting pathogen detection results (eg seropositive but PCR-negative individuals) should be described.

 Overall the manuscript is a scientifically robust and well-articulated protocol for developing a valuable global resource on arenavirus and hantavirus ecology.

Is the study design appropriate for the research question?

Yes

Is the rationale for, and objectives of, the study clearly described?

Yes

Are sufficient details of the methods provided to allow replication by others?

Yes

Are the datasets clearly presented in a useable and accessible format?

Yes

Reviewer Expertise:

Virology

I confirm that I have read this submission and believe that I have an appropriate level of expertise to confirm that it is of an acceptable scientific standard.

# Response

**Published:** 2025-08-05

**Authors:** David Simons

**Affiliations:** Department of Anthropology, The Pennsylvania State University, State College, PA, USA

Thank you for your constructive and detailed summary of our protocol. Your feedback has helped us clarify and improve several key aspects of our methodology. Following your suggestions, we have revised the manuscript to:

1. List objectives explicitly: We have added a bulleted list of study objectives in the introduction to improve clarity and readability.
2. Refine data processing: We have provided a detailed, reproducible set of decision rules for taxonomic harmonization, which will ensure consistency and transparency in how we handle ambiguous species names.
3. Clarify handling of conflicting results: We added a section to explain our approach to conflicting pathogen detection data. We will preserve all assay results, acknowledging that different tests capture distinct biological signals, and will maintain a one-to-many data structure between host and pathogen records.

These revisions directly address your points and significantly enhance the scientific rigor and transparency of our protocol.

# Reviewer report: Reviewer response for version 1

**DOI:** 10.21956/wellcomeopenres.26520.r122839

**Published:** 2025-05-28

**Authors:** Peter Daszak

**Affiliations:** 1 Nature.Health.Global., Inc., Tallman, New York, USA

Review of Simons et al. Project ArHa

 The paper describes a database to collate information on arenaviruses and hantaviruses. These viruses include some significant zoonotic threats to human health with high case loads (e.g. Lassa fever), and high mortality (e.g. Hantavirus pulmonary syndrome) and that are distributed widely (Asia, Africa, Americas). Despite this, data on the distribution of the viruses in their rodent reservoirs is fragmented, not systematically collected, and either privately held or deposited in a range of online databases. Likewise case reports and outbreak investigation data are often deposited in a non-systematic way, making it difficult to analyze distribution, assess biases in reporting and surveillance, and conduct predictive modeling. There are some notable exceptions, but these usually focus on single viruses, clusters of case reports over short time periods, or test specific hypotheses where the viral data often are scant compared to the environmental, demographic or other correlated data in the analysis (e.g. rodent hantaviral prevalence vs. climate, Lassa fever human cases vs. demography).

 There is huge utility for databases on zoonotic viruses that have wildlife reservoirs and are sensitive to environmental and demographic factors for their emergence. Arenaviruses and hantaviruses causes diseases that appear to have cycles of high prevalence (e.g. HPS in the Southeastern USA, Lassa fever in West Africa) and for which causal correlates are complicated to analyze and require large, multi-year datasets.

 The search protocols, data inclusion and exclusion criteria, data extraction and processing protocols are logical, and deal appropriately with all of the typical pitfalls that these data present (e.g. lack of consistency in sampling denominator reporting, species misidentification).

 The goal is very important and useful to global health, disease ecology, surveillance and predictive modeling, as well as to potentially anlayzing the drivers of spillover risk. The approach is valid. The key weaknesses that should probably be addressed in more detail are:

  

1. There may be substantial data on prevalence from papers in regional journals, published over a decade ago that may not be in PubMed or WoS
2. Many non-English language papers will be of relevance and an approach to include these may make the database more powerful
3. Large stretches of suitable habitat for potential reservoir rodent species may have had very little surveillance and this needs to be addressed, or perhaps should be one of the early studies from the database
4. Sampling biases are likely to: be substantial geographically and temporally, linked to ability to fund research and surveillance, and linked to availability of testing technology. It would be good to have a strategy to deal with some of this in the database design and also in the early research from the database

Is the study design appropriate for the research question?

Yes

Is the rationale for, and objectives of, the study clearly described?

Yes

Are sufficient details of the methods provided to allow replication by others?

Partly

Are the datasets clearly presented in a useable and accessible format?

Not applicable

Reviewer Expertise:

Emerging zoonotic diseases. Wildlife reservoirs of zoonoses. Viral emergence.

I confirm that I have read this submission and believe that I have an appropriate level of expertise to confirm that it is of an acceptable scientific standard, however I have significant reservations, as outlined above.

# Response

**Published:** 2025-08-05

**Authors:** David Simons

**Affiliations:** Department of Anthropology, The Pennsylvania State University, State College, PA, USA

Thank you for your valuable feedback. We agree that a comprehensive database must address the limitations of traditional search methods and sampling biases. In response to your comments, we have updated the protocol to: 1. Expand the search strategy: We will now conduct a second round of searches in regional databases such as African Journals Online (AJOL) and SciELO to capture literature not indexed in global databases. 2. Improve data inclusivity: Our protocol confirms we do not exclude non-English language papers and will use both automated tools and native speakers from our network for translation. 3. Address sampling bias: We have added a section detailing our plan to create a spatiotemporal raster of sampling effort. This will allow us to map surveillance gaps and enable more robust, bias-aware predictive modeling. These changes strengthen our methodology and ensure the resulting dataset will be a more accurate and representative resource for the research community.
